# An acute gastroenteritis outbreak associated with breakfast contaminated with norovirus by asymptotic food handler at a kindergarten in Shenzhen, China

**DOI:** 10.1186/s12879-021-05762-z

**Published:** 2021-01-12

**Authors:** Yuan Li, Xiangbo Fan, Guangqing Yu, Peinan Wei, Yong Wang, Hongxiong Guo

**Affiliations:** 1 Shenzhen Bao’an Center for Disease Control and Prevention, Shenzhen, China; 2 Hezhou Center for Disease Control and Prevention, Hezhou, China; 3 Wuqing Center for Disease Control and Prevention, Tianjin, China; 4 Department of Epidemiology, School of Public Health, Nanjing Medical University, Nanjing, China; 5 Jiangsu Provincial Center for Disease Control and Prevention, 172 Jiangsu Road, Nanjing, China

**Keywords:** Norovirus, Acute gastroenteritis outbreak, Asymptotic food handler, Kindergarten

## Abstract

**Background:**

An outbreak of acute gastroenteritis occurred in a kindergarten located Shenzhen City on March 4, 2018. We were invited to investigate to the risk factors associated with this outbreak.

**Methods:**

We conducted retrospective cohort-studies on three different groups of subjects in order to figure out the difference of incidence of acute gastroenteritis among subjects of different activities on March 2: group one consisted of people who attended the Lantern festival activities; group two consisted of children and employees who ate breakfast and bread provided by the kindergarten; and groups three consisted of children and employees who did not eat breakfast or bread provided by the kindergarten. Fecal, anal swabs, dishware swabs and hand swabs specimens were collected in the study. Bacteria known to cause acute gastroenteritis were cultured. Viruses associated with acute gastroenteritis were tested using real-time PCR. Capsid gene fragment of 557 bp of norovirus was amplified and sequenced. The phylogenetic tree was constructed with MEGA 7.0 using neighbor-joining method based on capsid gene fragment of norovirus.

**Results:**

A total of 143 suspected cases were identified in this outbreak. Diarrhea happened more often in adults than in children while emesis and bellyache were more frequently found in children than in adults. Higher AGE incidence was observed in group 2, children and employees who had breakfast in the kindergarten on March 2, as well as in group 3, and among employees who eating bread involved in breakfast provided on March 2. Five anal swab specimens were positive for norovirus. All noroviruses belongs to group II.3 and have an identity more than 99%.

**Conclusion:**

A chef, as an asymptomatic carrier with norovirus, was the infectious resource in this outbreak. He contaminated breakfast food provided on March 2. Although morning check is implemented in kindergartens of China, employees are often excluded in morning check. Our finding highlights the importance of morning check covering employees and periodical training for cooks.

**Supplementary Information:**

The online version contains supplementary material available at 10.1186/s12879-021-05762-z.

## Background

Norovirus (NoV) is a highly contagious agent that can infect individuals very easily. It transmits with ease through food, water, air and close contact. It was estimated that approximate 20% of all acute gastroenteritis (AGE) cases and about 200,000 deaths are caused by NoV each year in the world [1–3]. Foodborne norovirus-linked AGE is usually the result of consumption of food contaminated during food production or preparation [4]. Among foods, sea food and vegetable are predominant causes of outbreaks of foodborne norovirus-linked AGE [4].

**Fig. 1:**
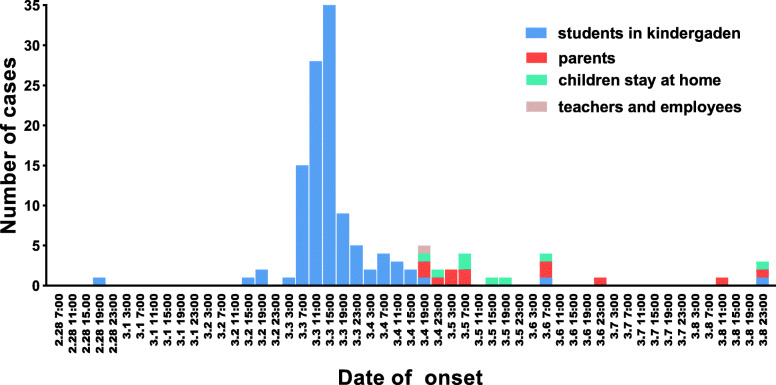
Date of onset of acute gastroenteritis symptoms observed in a kindergarten, Bao’an district, Shenzhen, China, March 2018

Neighboring Hong Kong, Shenzhen is a coastal city with a population of more than 10 million in Southern China. Among Shenzhen’s X administrative districts, the Bao’an district is the biggest in terms of area and population. In a kindergarten in Bao’an district, from 15:00 March 2, 2018, an increasing number of children showed symptoms of vomiting. The numbered peaked at 15:00 on March 3, 2018 (Fig. 1). The government agency, Shenzhen Bao’an Center for Disease Control and Prevention (SBCDC) responded immediately to identify the causes of this outbreak on March 4, 2018 in order to curtail the outbreak as soon as possible. From March 5 to 13, Three retrospective cohort studies to investigate the risk factors associated with this acute gastroenteritis outbreak. In this study, we will present epidemiological characteristics of this AGE outbreak.

## Methods

### Setting

**Fig. 2:**
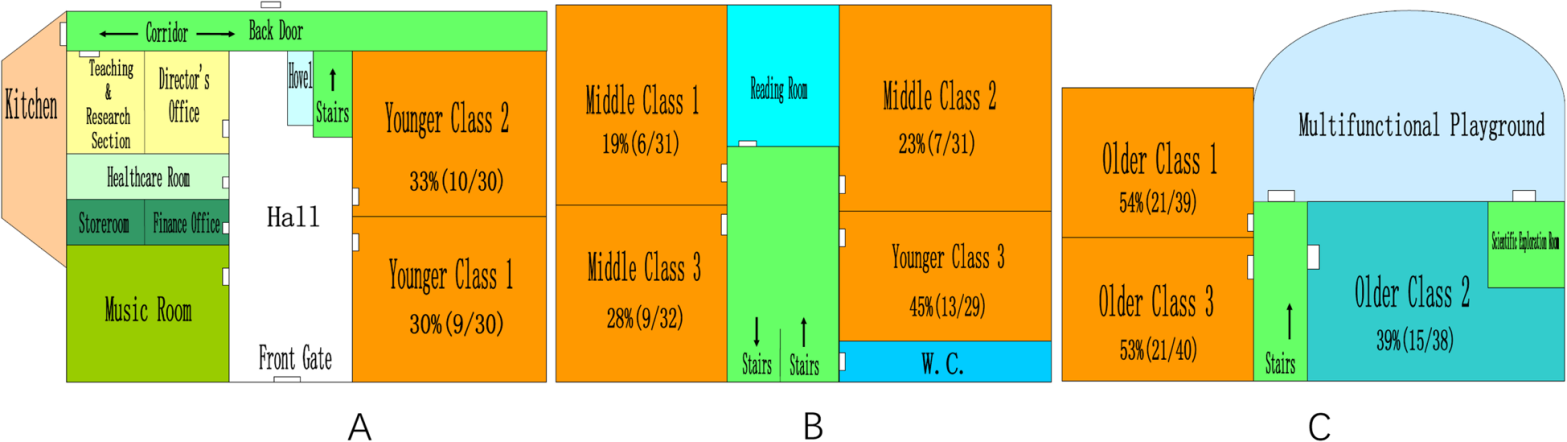
The diagram of the building in the kindergarten, Bao’an district, Shenzhen, China. **a**, **b** and **c** is the first, second, and third floor, respectively

The kindergarten has a three-floor building and a playground, as shown in Fig. 2. There are 300 children (138 males and 162 females, aged from 3 to 6) and 46 employees (30 teachers, 7 sanitation workers, 4 cooks, 3 managers and 2 security staff). Based on their age, the children are assigned into one of the three grades junior grade (3 to 4 years old), middle grade (4 to 5 years old) and senior grade (5 to 6 years old). There are three classrooms for each grade and every classroom has a separate washing room. The kindergarten offers breakfast and lunch for the children and employees. The food is made on site in its’ own facility. All people drink boiled city water.

### Epidemiology investigation

Children attending the kindergarten and the employees were included in the study, as well as children’s parents who attended a Lantern Festival party held on March 2 in the kindergarten. A subject with vomiting (more than once every 24 h) or diarrhea (more than three time every 24 h) after March 2, 2018 is defined as a suspect case. A suspect case that tested positive for norovirus by reverse transcription polymerase chain reaction (RT-PCR) is considered a confirmed case. A subject without symptom of AGE but norovirus positive in RT-PCR test is a virus carrier. Patients who did not go to the kindergarten during this outbreak were defined as secondary cases. A structured questionnaire was used to conduct epidemiology investigation (Supplement material 1). This study was approved by the Ethics Committee of SBCDC.

### The determination of exposure factors

On February 28, one child (child X) at the senior Class 3 reported vomiting and diarrhea. The child took medicine at home on March 1. Since the AGE cases were reported in all classrooms, it is not very likely that child X was the source of breakout. His younger sister at the Middle Class 3 took part in the Lantern party hosted by the kindergarten on March 2. In the party, the parents, children and teachers made dumplings, Tang-yuan, and ate the dumplings or/and Tang-yuan afterwards. It was proposed that virus transmitted through dumplings/Tang-yuan be a probable primary risk factor associated with this AGE outbreak. A retrospective cohort study was conducted to test this hypothesis. An epidemiology investigation was conducted by telephone interview of the party precipitants with a structured questionnaire. However, the investigation result shows that the hypothesis was false (as shown in Table 1).

**Table 1:** Univariate analysis of risk factors involved in Lantern Festival activity associated with the outbreak of acute gastroenteritis, in Bao’an district, Shenzhen, China, March 2018

Variable	Cases	controls	Chi-square	*P* values
Make dumpling			2.47	0.116
Yes	46 (54.1)	182 (64.3)		
NO	39 (45.9)	101 (35.7)		
Make Tang-yuan			1.46	0.228
Yes	57 (67.1)	167 (59.0)		
NO	28 (32.9)	116 (41.0)		
Eat dumping			0.02	0.885
Yes	29 (34.1)	92 (32.5)		
NO	56 (65.9)	191 (67.5)		
Eat Tang-yuan			1.14	0.286
Yes	45 (52.9)	129 (45.6)		
NO	40 (47.1)	154 (54.4)		

In this outbreak, onset of AGEs first appeared on children and employees and then on the parents attending the Lantern festival activities. It implied that only the children and employee cases shared the common exposure associated with this AGE outbreak, not the parents. Moreover, the children and employees made and ate dumplings/Tang-yuan in the morning while the outbreak occurred in the afternoon on the same day, there was simply no enough time for virus to incubate enough to a state of an outbreak of this size. Deducing by incubation time of norovirus infection, we found that the breakfast supplied in the morning on March 2 was very suspicious as it was shared by the children and employees. The second retrospective cohort-study was conducted to verify the hypothesis that food items of the breakfast supplied in the morning on March 2 be associated with this outbreak. As the investigation goes on, it was discovered that bread was the only item shared by the children and employees. To verify whether this outbreak was caused mainly by the contaminated bread or not, the third cohort-study was conducted on the employees to eliminate potential recall bias in the young children.

#### Laboratory tests

The collection of specimens is shown in Table 2. All samples were transported to SBCDC for microorganism test. Thirty-six samples were collected on March 4, and tested for *Salmonella*, *Shigella*, *Proteus*, *Campylobacter jejuni*, *Staphylococcus aureus*, *E. coli*, *Streptococcus*, *Bacillus cereus*, *Vibrio Parahaemolyticus,Vibrio cholerae*, *Listeria monocytogenes*, norovirus and *sapovirus*. Four feces of the children and 2 anal swabs of the chefs were collected on March 14. The anal swabs of the 3 children whose anal swabs specimens collected on March 4 were collected on March 26 again. Only norovirus and *sapovirus* were tested for the samples collected on March 14 and 26 using RT-PCR methods.

**Table 2:** The date of the specimens collected from this outbreak in the kindergarten in Bao’an district, Shenzhen, China, March 2018

Collection date	Specimen (n)						
	Food	Tableware swab	Hand swab of Chef	Anal swab of Chef^a^	Anal swab of children	Vomitus	Fecal of children
March 4	10	10	6	5	5	–	–
March 14	–	–	–	2^d^	–	–	4^b^
March 26	–	–	–	1^f^	3^c^	–	–

^a^asymptomatic

^b^3 of 4 were positive for norovirus, and they were 3 years old, and two in younger class one and one in younger class two

^c^2 of 3 were positive for norovirus, and both of them are 3 years old, and on in younger class one and the other in younger class two

^d^one of two samples was collected from Chef Nei, and Nei’s sample was positive for norovirus

^f^this sample was collected from Chef Nei and positive for norovirus

#### The culture and identification of bacteria

All specimens from the AGE patients were screened for bacteria by conventional biochemical methods in SBCDC. All isolates were identified using the Rapid ID32E strips (bioMérieux Corp., Singapore) on an automatic biochemistry analyzer (Hitachi 917; Boehringer Mannheim, Japan).

#### RNA extraction and amplification

Approximately 50–80-μg stool samples were weighed, diluted 1:10 in nuclease-free water, and then vortexed for 30 s. Samples were clarified by centrifugation at 6800 ×g for 10 min at room temperature. Viral RNA was extracted from 200-μl processed samples using a High Pure Viral Nucleic Acid Kit (Roche, Schweiz) according to the manufacturer’s instructions. A Real-time fluorescence PCR for sapovirus and norovirus was performed using an ABI Quant Studio 6 Flex real-time PCR system with a commercial kit (Takara, Japan) according to the manufacturer’s instructions. The Primer sets MON431 (TGGACIAGRGGICCYAAYCA) and G2-SKR (CCRCCNGCATRHCCRTTRTACAT) were used to amplify capsid gene fragment (557 bp) according to previous description [5]. PCR product was sequenced at Takara Bio INC.

#### Phylogenetic analysis

The sequences were manually edited using the BioEdit software, then analyzed using the MEGA 7.0 software. The reference strains include the reference strains of GII.3 isolated from other places, the sequences of G II.4 was used to as an outgroup. The phylogenetic tree was constructed using neighbor-joining method with 1000 bootstrap replications. GenBank accession numbers are from MT093374 to MT093377.

### Statistical analysis

Attack rate was calculated as the proportion of cases among the targeted students in the kindergarten. Comparisons of categorical data were performed using Pearson’s chi-square test or Fisher’s exact test. The odds ratio (OR) was calculated. A *p*-value of < 0.05 was considered statistically significant. All statistical analyses were performed with R 3.5.0 software (R Foundation for Statistical Computing, Vienna, Austria).

## Results

### Descriptive epidemiology

A total of 143 suspected cases were identified in this outbreak, including 20 adults (8 employees and 12 parents) and 123 children (13 family cases, and 110 attending children). Ninety-four percent (94%) of the cases reported vomiting, 69% bellyache, 57% fever and 30% diarrhea. Among adults with AGE, 50% of them had bellyache, 50% had fever, 75% had diarrhea and 60% had emesis. Among children with AGE, 72.1% had bellyache, 56.8% had fever, 75% had diarrhea and 99.2% had emesis. Fever showed up more often in adults than in children while emesis and bellyache showed up more frequently in children than in adults (Table 3).

**Table 3:** The difference of clinical features between adults and children in this acute gastroenteritis outbreak in Bao’an district, Shenzhen, China, March 2018

Characteristic	Adults n(%)	Children n(%)	χChi-square	*p* value
Bellyache			114.34	<0.001
Yes	10 (50)	88 (72.1)		
No	10 (50)	34 (27.9)		
Fever			66.13	<0.001
≥ 38 °C	2 (10)	43 (34.9)		
< 38 °C	8 (40)	27 (21.9)		
No	10 (50)	47 (43.2)		
Diarrhea			–	0.37^a^
≥5	5 (25)	1 (0.8)		
<5	10 (50)	27 (22.1)		
No	5 (25)	94 (77.0)		
Emesis			–	<0.001^a^
≥ 10	1 (5)	21 (17.2)		
5~10	5 (25)	38 (31.1)		
< 5	6 (30)	62 (50.8)		
No	8 (40)	1 (0.8)		

Five child has no respond

^a^Fisher’s exact test

The first case appeared at 15:00 on March 2 while number of cases peaked between 11:00–15:00 on March 3. The epidemic curve shows that it is a common source outbreak followed by the secondary transmission within families (Fig. 1). Suspected cases were reported on children in all the nine classrooms and the employees (as showed in Fig. 2). The incidence of suspected cases in these classrooms ranged from 19 to 54%. The secondary cases occurred within the families. The incubation time ranged from 9 h to 59 h, with a median of 31 h. Twenty-four secondary cases were reported with 12 child incidences and 12 adult incidences.

### Risk factors for this AGE outbreak

In the retrospective cohort study, no significant difference of AGE incidence was observed between group 1 and group 2, that is, those who made and ate dumplings (as shown in Table 1). In the secondary retrospective cohort study, we compared 206 subjects that had breakfast in the kindergarten and 13 subjects that did not have breakfast there. Significant difference of AGE incidence between two groups was observed (chi-square value is 6.1 and *P* = 0.01, as shown in Table 4). The third retrospective cohort study showed that there was higher incidence of AGEs among employees that ate bread than among those did not eat bread in the morning of March 2(shown Table 5).

**Table 4:** The contribution of eating breakfast in the kindergarten to onset of AGE in this outbreak in the kindergarten in Bao’an district, Shenzhen, China, March 2018

Exposure	AGE Cases	non-AGEs	RR (95%CI)	*P* value
Eating breakfast in kindergarten	108	98	6.1 (1.3–28)	0.01
No	2	11

**Table 5:** The contribution of eating bread on incidence of acute gastroenteritis in this outbreak in the kindergarten in Bao’an district, Shenzhen, China, March 2018

Eating bread	Cases	The healthy	χ2	P	RR
Yes	7	22	2.69	0.041	1.318 (1.074~1.619)
No	0	15			

#### The results of bacteria culture and nucleic acid test for viruses

All samples collected on March 4 tested negative for bacteria, norovirus and sapovirus. Out of the specimens collected on March 14, three fecal specimens from two children and one anal swab from Chef Nei tested positive for norovirus using PCR test. Further analysis revealed that the genotype of the norovirus was GII.3. Among the specimens collected on March 26, 2 of the 3 anal swab specimens of children and one anal swab specimen of Chef Nei tested positive for norovirus.

#### Phylogenetic analysis

**Fig. 3:**
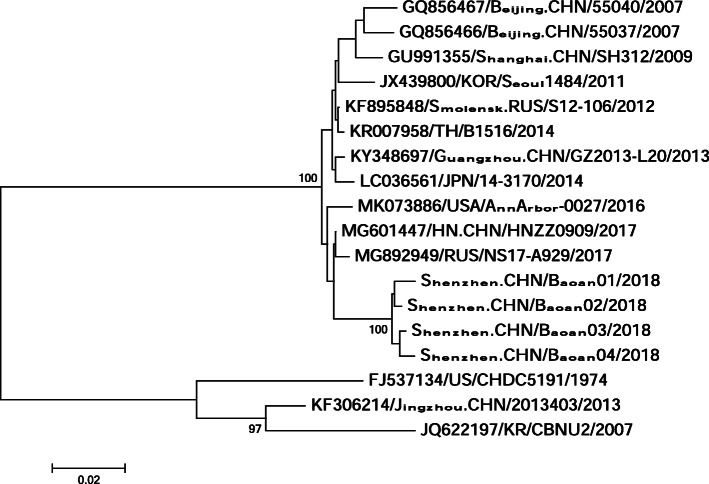
Phylogenetic analysis based on norovirus VP1 gene sequences using neighbor-joint methods

Out of the six real fluorescence time PCR positive for norovirus, four successfully amplified for genotype identification. In Fig. 3, the sequence labeled as Shenzhen.CHN/Baoan04/2018 is Chef Nei’s. As showed in Fig. 3, norovirus in this outbreak can be assigned to genotype groupII.3, and they clustered together and formed a monophyly with a more than 99% identity.

### The bread making and distribution

The bread involved in this event was made by Chef Nei in the afternoon of March 1. There was no one else involved in the whole process, from making to distribution. From scratch, the steps required to make bread includes mixing, stirring, kneading, toasting, and slicing. All bread was toasted 35 min twice. At 6:30 in the morning of March 2, the bread was reheated for 5 min, sliced and then placed in dishes. Then the bread dishes were covered by food wrapping film. During breakfast time, teachers took bread to the classrooms and distributed the break to the children.

#### Control measures

The following measures were taken: (1) Classes were suspended from March 3 to 5. After classes were resumed, routine checks were conducted in the morning and at noon. All suspected cases were sent to doctors immediately and quarantined if confirmed. (2) Chef Nei was not released from quarantine until anal swab specimen tested negative for norovirus. (3) Instruments and the facility were thoroughly cleaned and disinfected When classes were resumed, air in the classroom was ventilated timely. (4) All employees including chefs and teachers were required to have morning checkups every day. (5) Re-education of food handling safety for all employees.

## Discussion

Our results reveals that the breakfast on March 2 was the infectious source of this AGE outbreak caused by GII.3 noroviruses. Bread shows high relative risk (1.318, 95%CI,1.074–1.619). Although the specimen taken from the bread tested negative for norovirus, phylogenetic analysis shows that all sequences in this outbreak form a monophyly (Fig. 3), which is a strong evidence that all cases involved this outbreak share a common infectious source. Among 4 chefs, only Chef Nei tested norovirus positive and took part in cooking all food items provided in the morning of March 2. With all these findings we are confident to draw the conclusion that this AGEs outbreak was caused by food contamination by an asymptomatic food handler carrying noroviruses.

The asymptomatic food handlers are assumed to have played an important role in the transmission of norovirus [6–11]. When cooking food, norovirus may be transmitted through finger, fomites, air. In most cases, the asymptomatic cook(s) often do not realize that he/she has contaminated food. As in this study, Chef Nei thought that he had been kept a good hand hygiene practice during the whole process of making bread. Among foodborne AGE outbreaks, sea food and salad contaminated with norovirus are the most common [4, 12, 13]. Up to now, very few norovirus outbreaks were associated with contaminated bread. This may be partially attributed to the virus often killed by the high temperature when baked. However, bread is still vulnerable to contamination during slicing and distribution.

The long shedding time of norovirus in asymptomatic carrier and patients with mild symptoms present a huge challenge to prevention of norovirus epidemic [7, 14]. As we knew, the shedding time of person with norovirus infection range from several days to 2 months [7, 15]. A high proportion of patients with norovirus present mild symptoms and more than 30% of them are asymptotic [16]. Therefore, to prevent AGE outbreak caused by norovirus, good hand hygiene practice is very important for all food handlers. All staffs working in food facilities in kindergartens and schools should be trained periodically and routinely, especially in enterovirus epidemic seasons.

In China, all kindergartens are required to perform daily morning checkups on all children before they are allowed to enter the kindergarten. This measure keeps the children with potential infectious disease away from kindergarten and stops them as being infectious source. However, teachers and other employees are normally excluded from daily checkups. Among AGE outbreaks caused by norovirus, foodborne transmission has been one of the major routes, and food handlers have usually been identified as the infectious source. Therefore, morning checkups should be required for all employees working in kindergartens. Moreover, to prevent foodborne disease outbreak, the food handling safety training should be conducted regularly for all facility staffs.

In China, GII.4 has been the main genotype associated with viral AGE [17]. In recent years, GII.17 emerged and caused epidemic frequently, and even became the main genotype circulating in some places [18, 19]. AGEs caused by GII.3 have been reported in Peking, Guangzhou, Zhengzhou, Shanghai since 2009 [20]. It is unknown if GII.3 would be one of the main genotypes in the future along with GII.17. The complexity and constantly changing nature of noroviruses in a region is a challenge to the development of vaccine against norovirus. AGE surveillance system was established in some regions, but molecular surveillance has not been implemented. Our finding highlights the importance of molecular surveillance again.

There were some limitations in this study. Firstly, no vomitus specimens of patients in this outbreak were collected. Secondly, the retrospective investigations were conducted relatively late, which may increase the bias of information recalled by the employees. Finally, to avoid recall bias among children, only teachers and employees were enrolled into a further cohort-study to identify association of the bread with the incidence of AGE.

## Conclusions

The asymptomatic food handlers carrying norovirus caused AGE outbreak in public places including kindergarten. To prevent AGE outbreak associated with norovirus, food handlers should take daily checkups and receive periodic food safety trainings.

## Supplementary Information

**Additional file 1.** Breakfast Survey on March 2 (Lantern Festival)

## Data Availability

The datasets used in this study are available from the corresponding author on reasonable request.

## Abbreviations

NoV: Norovirus
AGE: Acute gastroenteritis
SBCDC: Shenzhen Bao’an Center for Disease Control and Prevention
