# Supplementary material for: An acute gastroenteritis outbreak associated with breakfast contaminated with norovirus by asymptotic food handler at a kindergarten in Shenzhen, China
Source: BMC Infect Dis. 2021 Jan 12;21:54. doi: 10.1186/s12879-021-05762-z (PMC7802125; doi:10.1186/s12879-021-05762-z)
Supplement: Supplementary file 1 — Additional file 1. Breakfast Survey on March 2 (Lantern Festival) [file 12879_2021_5762_MOESM1_ESM.docx]

**Breakfast Survey on March 2 (Lantern Festival)**

1. Name： Telephone: _________________
2. Gender：□ Male □ Female
3. Nationality：□ Han □ Others，Please describe in detail：
4. Birthday： Year Month Day
5. Profession: _________

① Theacher ② Student ③ Cafeteria staff

1. Onset between March 2 and March 5: □Yes □No

(Answer "No", please skip to Question 10)

1. Date of onset： Year Month Day
2. Vomit: □Yes □No If yes, Times____day
3. Diarrhea: □Yes □No If yes, Times____day

10.Whether to have breakfast in kindergarten on March 2: □Yes □No

If yes, Food type □ bread Intake____  □ rice flour Intake____

□ Sour beans Intake____ □porridge Intake____

11. Are other members of the family affected: □Yes □No

If yes, date of onset： Year Month Day

Name of investigator： Date of investigation：
